# A bidirectional Mendelian randomization study investigating the causal role between gut microbiota and insomnia

**DOI:** 10.3389/fneur.2023.1277996

**Published:** 2023-12-07

**Authors:** Jie Yang, Tengfei Su, Yating Zhang, Menghan Jia, Xiang Yin, Yue Lang, Li Cui

**Affiliations:** ^1^Department of Neurology, The First Hospital of Jilin University, Changchun, China; ^2^Department of Otolaryngology, The Second Hospital of Jilin University, Changchun, China

**Keywords:** gut microbiota, insomnia, Mendelian randomization, causal inference, GWAS

## Abstract

**Background:**

It has emerged that disturbances of the gut microbiota (GM) are linked to insomnia. However, the causality of the observed associations remains uncertain.

**Methods:**

We conducted a two-sample Mendelian randomization analysis based on genome-wide association study data to explore the possible causal link between GM and insomnia. The GM data were from the MiBioGen consortium, while the summary statistics of insomnia were obtained from the FinnGen consortium R9 release data. Cochran’s Q statistics were used to analyze instrumental variable heterogeneity.

**Results:**

According to the inverse variance weighted estimates, the family Ruminococcaceae (odds ratio = 1.494, 95% confidence interval:1.004–2.223, *p* = 0.047) and the genus Lachnospiraceae (odds ratio = 1.726, 95% confidence interval: 1.191–2.501, *p* = 0.004) play a role in insomnia risk. In contrast, the genus Flavonifractor (odds ratio = 0.596, 95% confidence interval: 0.374–0.952, *p* = 0.030) and the genus Olsenella (odds ratio = 0.808, 95% confidence interval: 0.666–0.980, *p* = 0.031) tended to protect against insomnia. According to the reverse MR analysis, insomnia can also alter GM composition. Instrumental variables were neither heterogeneous nor horizontal pleiotropic.

**Conclusion:**

In conclusion, our Mendelian randomization study provides evidence of a causal relationship between GM and insomnia. The identified GM may be promising gut biomarkers and new therapeutic targets for insomnia. This investigation also provides a foundation for future studies examining the influence of GM on sleep disorders beyond insomnia, with potential implications for redefining the mechanisms governing sleep regulation.

## Introduction

Insomnia represents a common sleep disorder characterized by challenges falling or remaining asleep, poor sleep quality, and diminished daytime functionality (1, 2). Evidence suggests that insomnia may be associated with various medical conditions such as depression, diabetes, and high blood pressure among others. The etiology of insomnia may be linked to genetic, biochemical, neuroendocrine, immune, and psychosocial factors.

While traditional sleep research often centers around neurological factors, recent research reveals that gut microbiota (GM) may contribute to the development of the central nervous system (CNS) and affect the pathogenesis and progression of a variety of brain conditions. This bidirectional relationship is commonly referred to as the brain–gut axis. Growing empirical data demonstrates that GM and sleep interact through metabolites, the immune system, and neural pathways (3). Insomnia is associated with an altered GM in both acute and chronic insomnia patients (4). Reversal of GM imbalance has been shown to improve sleep duration and sleep efficiency (5). Based on the gut–brain axis—a dynamic bidirectional communication system, this study further clarified the causal relationship between insomnia and GM.

Although randomized controlled trials offer reliable methods of inferring causality, the use of GM as an intervention has limitations, incurring time, and expense. Observational studies are prone to various biases, hence the need for an alternative approach. Mendelian randomization (MR), which utilizes single nucleotide polymorphisms (SNPs) as instrumental variables, provides a means of making causal inferences independently from confounding factors and biases (6). As a result, MR seems more appropriate for epidemiological studies (7). Genome-wide association study (GWAS) data on GM and insomnia offer a foundation for our MR approach. Our study employs a two-way MR analysis for the first time, exploring the association between GM and insomnia to develop our understanding of such an association and offer new treatment possibilities.

## Methods

### Study design

**Figure 1:**
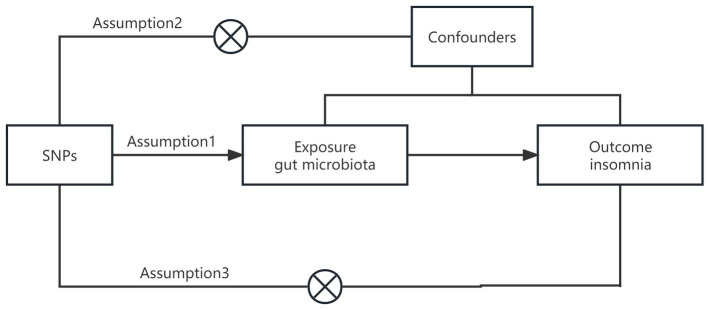
Graphical representation of the MR assumptions.

In this study, we applied a two-sample MR analysis to the pooled dataset of genome association studies to assess the causal relationship between GM and insomnia. In order to test the reliability of the results, a sensitivity analysis was performed. In order to satisfy the core assumptions of association, independence, and exclusivity of MR studies, the following core assumptions must be met: (1) instrumental variables must be strongly correlated with exposure factors; (2) instrumental variables cannot be correlated with any confounding factors that are associated with exposure and outcome; (3) instrumental variables can only influence outcome variables through exposure factors (8) (Figure 1). The content of this article has been checked against the STROBE-MR checklist (9).

### Data sources

Using the largest genome-wide meta-analysis of GM composition published to date by the MiBioGen consortium, the genetic variants of the GM were determined (10). Among 18,340 participants from 24 cohorts in 10 countries, including the United States, Canada, Israel, Korea, Germany, Denmark, the Netherlands, Belgium, Sweden, Finland, and the United Kingdom, the study analyzed 16S ribosomal RNA gene sequencing profiles and genotyping results. The GWAS summary statistics of insomnia were derived from the FinnGen consortium’s R9 release (*N* = 217,855) (11).

### Instrumental variable

An instrumental variable (IV) associated with GM was identified using a two-step approach in this study. To begin with, we selected SNPs that were significantly associated with GM at the genome-wide level (*p* < 1.0 × 10^–5^, R^2^ < 0.001, clumping window size = 10,000 kb) and were not associated with outcome. The final SNPs that were significantly associated with GM were obtained as instrumental variables through a query to the Phenoscanner database.

The F statistic was calculated to determine whether the selected instrumental variables have weak instrumental variable biases; *F* > 10 indicates that the correlation hypothesis cannot be tested further as there is no weak instrumental variable (12). F = beta^2^/se^2^, where beta represents the estimate and se represents the standard error of the effect allele on exposure (13).

### Mendelian randomization analyses

In this study, we employed an array of methodologies, including inverse variance weighting (IVW), MR-Egger regression, weighted median estimator, simple mode, and weighted mode, to ascertain a causal relationship between GM and insomnia. IVW was used as the primary MR method, and the results produced served as the main factor in determining whether there was a causal relationship. IVW combines Wald estimates for each SNP to deduce the impact of GM on insomnia. The outcomes derived from IVW remain unbiased, provided there is an absence of horizontal pleiotropy (14). MR-Egger regression can determine whether genetic variants have pleiotropic effects on outcomes that differ from zero (directional pleiotropy), as well as provide a consistent estimate of the causal effect under a weaker assumption (15). Despite 50% of genetic variation violating the MR assumption, the weighted median estimation method accurately calculates causal association effects (16). All outcomes were tested for significant horizontal pleiotropy using MR-PRESSO (17) and MR-Egger intercept regression to minimize the bias caused by it. Further examination of the association between GM and insomnia was conducted with four other established MR methods, including MR-Egger regression, weighted median estimator, simple mode, and weighted mode. To evaluate the impact of a single SNP on the results, we performed a leave-one-out analysis by removing each instrumental variable. Further tests of heterogeneity were performed for statistically significant results, including Cochran’s Q statistic, to ensure robustness. Statistics showed a significant difference at a *p*-value of <0.05. Moreover, we performed a reverse MR analysis between GM and insomnia, with selection criteria for SNPs of *p* < 5.0 × 10^–6^. Methodology and context are consistent with the forward model. We used the R packages two-sample MR (18) and MR-PRESSO (17) to carry out all statistical analyses.

## Results

**Figure 2:**
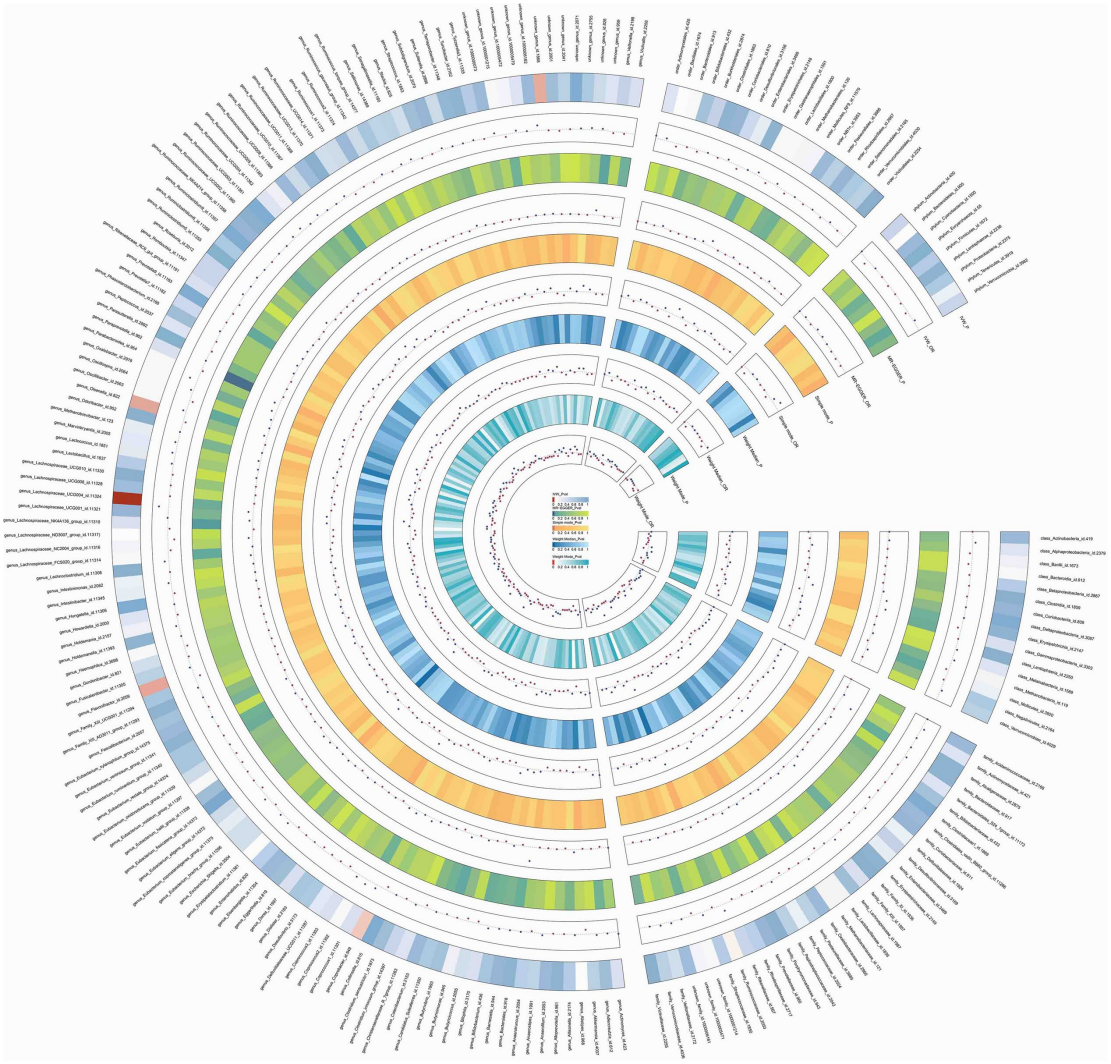
Circos of causal effects of GM on Insomnia. The first circle shows the name of GM, and the second to eleventh circles show the results of MR analysis, with the value of *p* magnitude indicated by color and the relevant annotation in the very middle of the figure.

**Figure 3:**
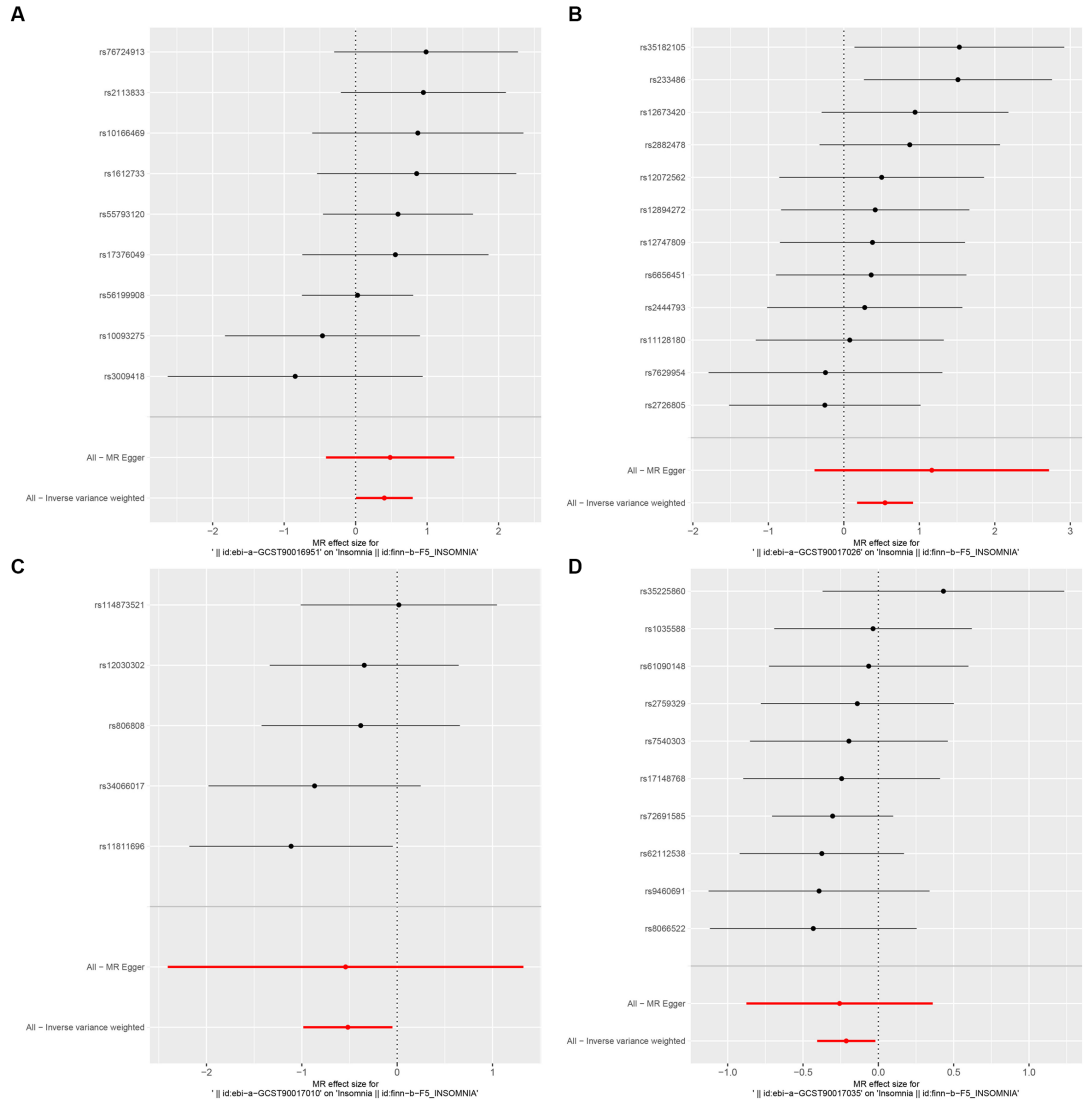
Forest plot of MR analysis of the causal relationship between GM and Insomnia. The x-axis shows the MR effect size of GM on insomnia. The y-axis shows the results of the analysis for each SNP. (**A**: family Ruminococcaceae; **B**: genus Lachnospiraceae UCG004; **C**: genus Flavonifractor; **D**: genus Olsenella.)

**Figure 4:**
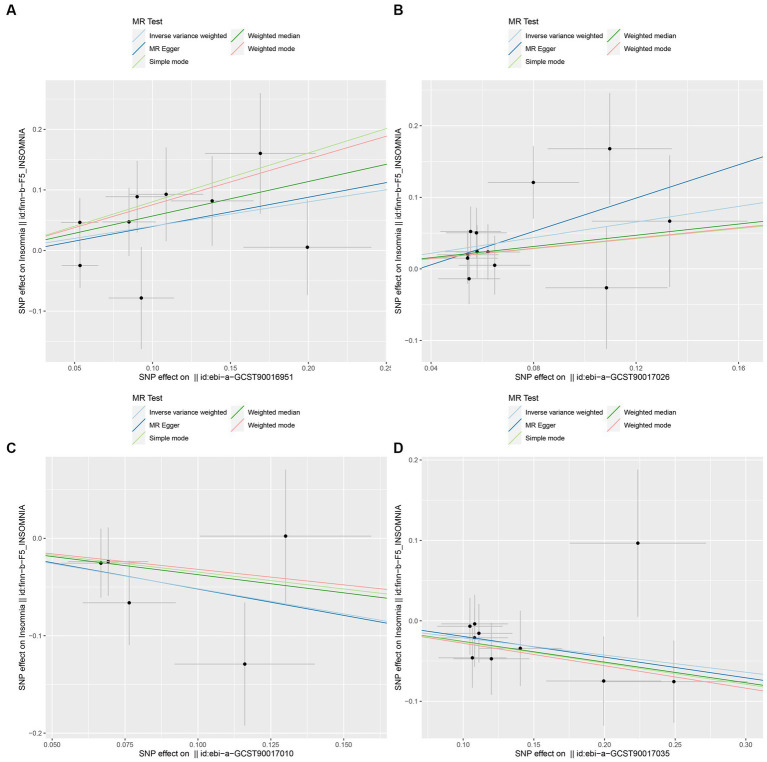
Scatter plot to visualize the causal effect of GM on insomnia. The slope of the straight line indicates the magnitude of the causal association. (**A**: family Ruminococcaceae; **B**: genus Lachnospiraceae UCG004; **C**: genus Flavonifractor; **D**: genus Olsenella.)

**Figure 5:**
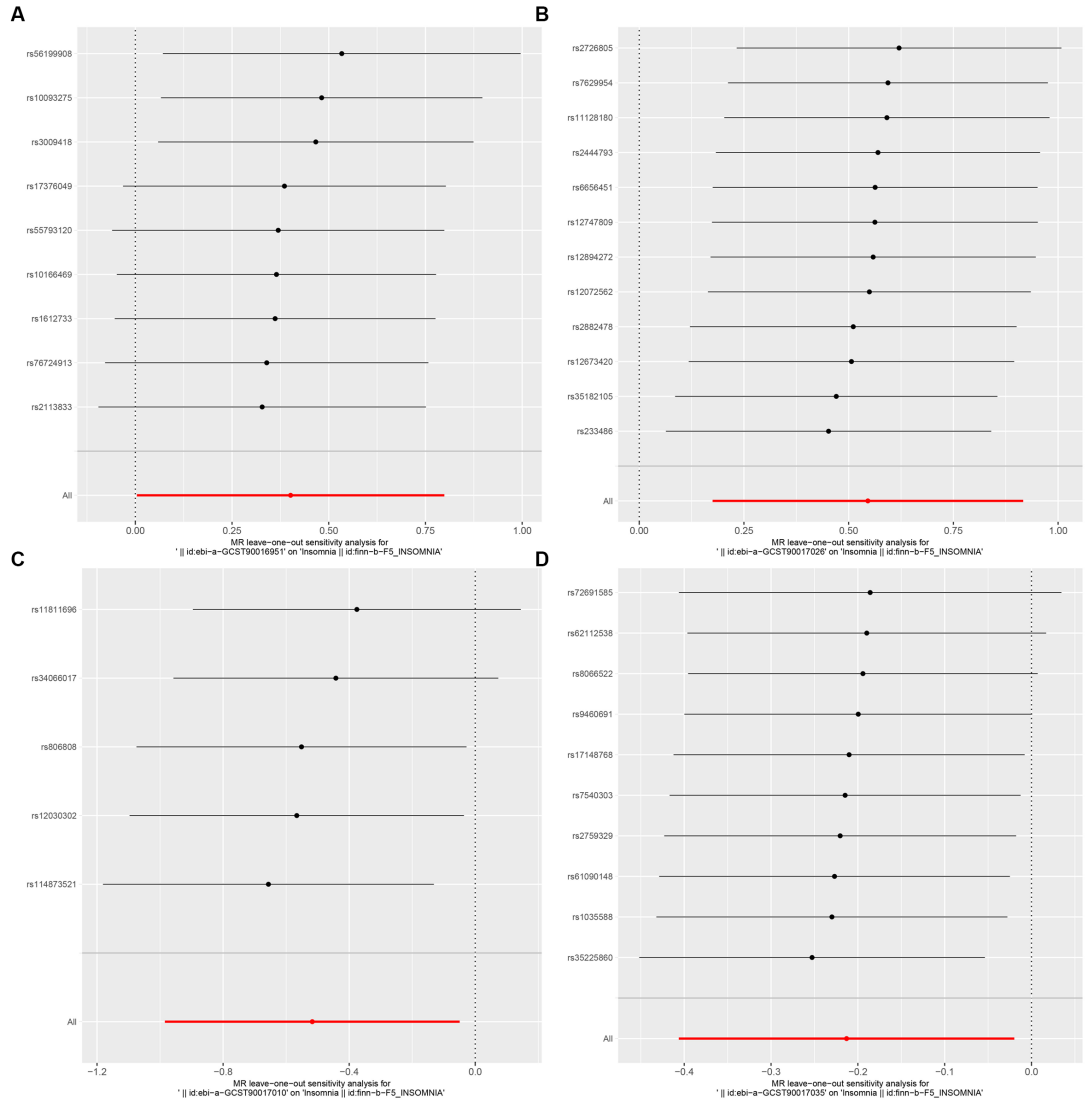
MR leave-one-out sensitivity analysis to assess the robustness of the causal relationship between GM and Insomnia. Circles indicate MR estimates for GM on insomnia using the fixed-effect IVW method if each SNP was omitted in turn. (**A**: family Ruminococcaceae; **B**: genus Lachnospiraceae UCG004; **C**: genus Flavonifractor; **D**: genus Olsenella.)

**Figure 6:**
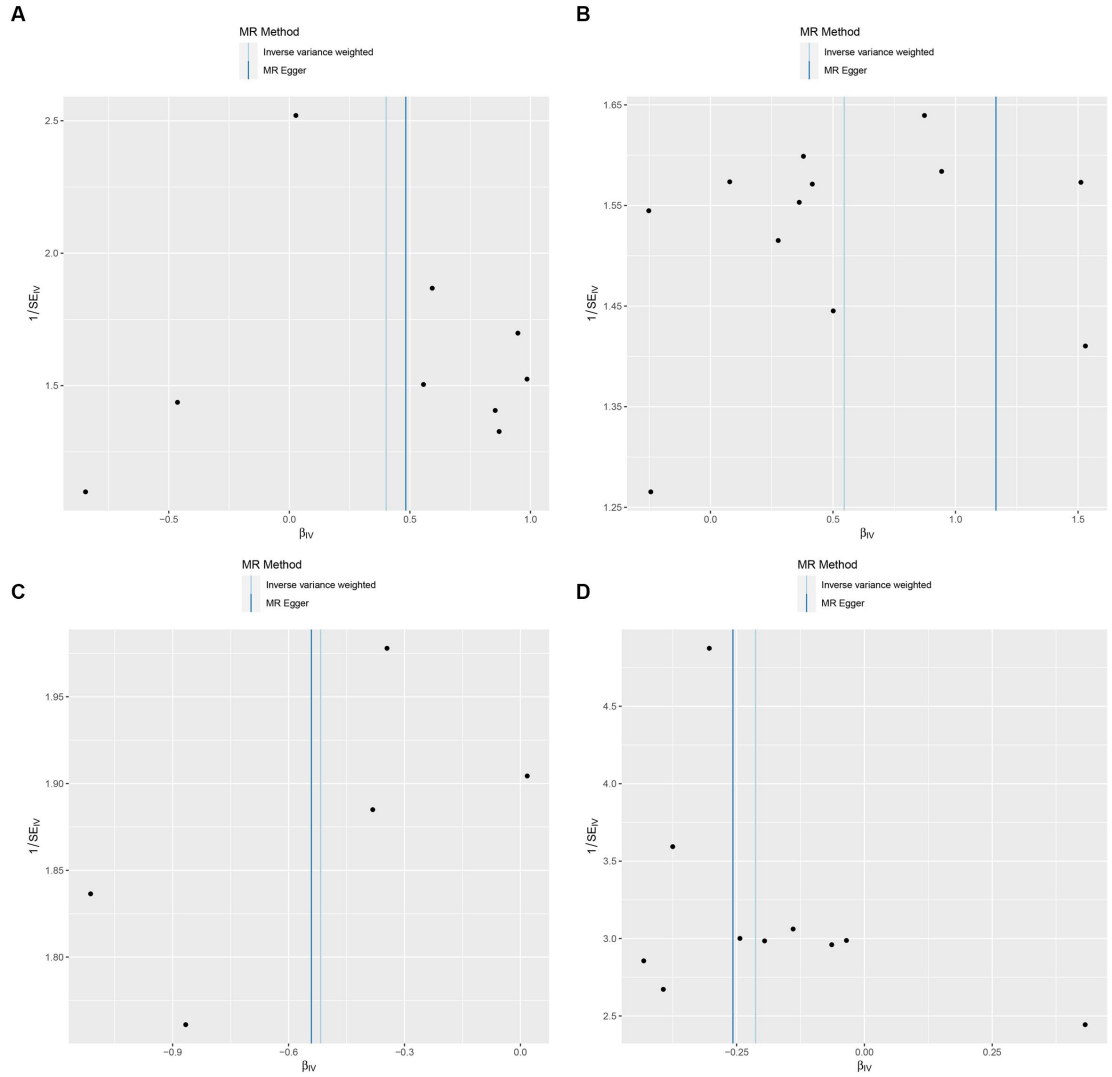
Funnel plot of the causal relationship between GM and Insomnia as assessed by MR analysis. (**A**: family Ruminococcaceae; **B**: genus Lachnospiraceae UCG004; **C**: genus Flavonifractor; **D**: genus Olsenella.)

In this study, upon excluding IVs exhibiting linkage disequilibrium from the GWAS dataset, we selected 2,270 SNPs associated with GM as IVs in this study (Supplementary Table S1, Sheet 1). The *F*-values of all SNPs included in the analysis were greater than 10, indicating no weak bias and ensuring accurate results. There was at least one MR method that showed a link between insomnia and four known bacteria genera, including family Ruminococcaceae family, genus Lachnospiraceae, genus Flavonifractor, and genus Olsenella (Figure 2). According to the inverse variance weighted method (Table 1; Figure 3), the family Ruminococcaceae (odds ratio = 1.494, 95% confidence interval:1.004–2.223, *p* = 0.0477) and the genus Lachnospiraceae (odds ratio = 1.726, 95% confidence interval: 1.191–2.501, *p* = 0.003) were SE risk factors, whereas genus Flavonifractor (odds ratio = 0.596, 95% confidence interval: 0.374–0.952, *p* = 0.030) and genus Olsenella (odds ratio = 0.808, 95% confidence interval: 0.666–0.980, *p* = 0.031) provided protection against insomnia. MR-Egger regression, weighted median estimator, simple mode, and weighted mode produced similar results (Figure 4). MR-PRESSO and MR-Egger intercept regressions were also used to test the stability of the aforementioned outcomes (Supplementary Table S1, Sheet 4), but no potential pleiotropy was detected. The results of the leave-one-out sensitivity analysis showed that the estimated effects of each SNP locus were not attributed to any one locus (Figure 5). In the included SNP effects, Cochran’s Q statistic showed no significant heterogeneity (Supplementary Table S1, Sheet 4). Our approach was supported by the funnel plot (Figure 6).

**Table 1:** Gut microbiota on insomnia result.

Exposure	No. SNP	IVW			MR-PRESSO	MR-Egger intercept regression	Cochran’s Q statistic
		Beta	*P*	OR (95%CI)	*P*	*P*	*P*
Family Ruminococcaceae	9	0.401	0.048	1.494 (1.004–2.223)	0.572	0.848	0.439
Genus Lachnospiraceae UCG004	12	0.546	0.004	1.726 (1.191–2.501)	0.716	0.440	0.683
Genus Flavonifractor	5	−0.517	0.030	0.596 (0.374–0.952)	0.648	0.981	0.594
Genus Olsenella	10	−0.213	0.031	0.808 (0.666–0.980)	0.903	0.886	0.899

The identical MR method was used to investigate the causal relationship between insomnia and GM in reverse analysis (Supplementary Table S2; Supplementary Figures 1–3). The presence of reverse causality was not observed in the reverse MR analysis. Insomnia was associated with a higher relative abundance of genus Eggerthella (Beta ± SE, 0.158 ± 0.066; *p* = 0.017) and a lower relative abundance of NB1n (Beta ± SE, −1.492 ± 0.065; *p* = 0.024) and genus Holdemanella (Beta ± SE, −0.139 ± 0.055; *p* = 0.012).

## Discussion

This is the first MR study to reveal a causal relationship between gut flora and insomnia based on GWAS data. This study confirmed that the family Ruminococcaceae and the genus Lachnospiraceae UCG004 are risk factors for insomnia, while the genus Flavonifractor and the genus Olsenella have potential protective effects on insomnia.

Over the past few decades, significant headway has been made in comprehending the bidirectional interactions linking GM and sleep, which happens through the brain–gut axis (3). First, a diverse range of metabolites produced by the gut and endocrine factors actively participate in sleep regulation. The neurotransmitters such as melatonin (19) and gamma amino butyric acid (GABA) among others synthesized in the intestine facilitate sleep initiation and reduce anxiety and stress while regulating emotions. On the other hand, neurotransmitters such as serotonin, orexin (20), and histamine (3) promote wakefulness. Microbial fermentation of dietary fiber in the intestine produces a considerable number of short-chain fatty acids (SCFAs) (21). Current research suggests that propionate has varying effects on sleep at different ages (22, 23). Butyrate may act as a sleep-inducing signaling molecule (24). Second, not only GM can send signals directly to the brain through the vagus nerve (25), but neurotransmitters produced in the gut can also do the same (26). The two-way link between GM and sleep can also be achieved by regulating the immune system (27, 28). Significantly, there is a well-understood link between GM and insomnia. For instance, Liu et al. demonstrated noteworthy fluctuations in GM composition and diversity in patients with insomnia relative to 10 healthy controls (29). Li et al. also validated that the GM of acute and chronic insomnia patients was marked by lower microbial richness and diversity, a reduced number of anaerobes, and short-chain fatty acid (SCFA)-generating microorganisms, while showing an increase in the number of potential pathogens compared to their healthy counterparts (4). Additionally, studies have asserted that gut microbes have an impact on brain function. A regional homogeneity study found a negative correlation between the relative abundance of lactic acid bacteria in the left fusiform gyrus and regional homogeneity values in patients with chronic insomnia (30). Moreover, treatment for the imbalance in the patient’s intestinal flora using antibiotics proved effective in improving sleep duration and efficiency while also regulating mood (5).

Existing studies on the impact of family Ruminococcaceae on sleep are inconclusive. Some investigations have associated chronic disruption of host circadian rhythm and sleep deprivation with an increase in family Ruminococcaceae levels, leading to microbial imbalance (31, 32). However, Zhou et al. found a significant reduction in the family Ruminococcaceae in patients with insomnia by comparing the intestinal microbiota composition of 24 patients with insomnia and 22 healthy controls (33). This study suggests that the family Ruminococcaceae is a risk factor for insomnia. Family Ruminococcaceae consists of a large number of Firmicutes in the intestinal environment that break down substrates that the host cannot digest, then ferment and convert these compounds into short-chain fatty acids (mainly acetate, butyrate, and propionate) that can be further absorbed by the host (34). This could be a probable leading cause of family Ruminococcaceae’s effect on patients’ sleep. As it turns out, family Ruminococcaceae has also featured in several inflammatory diseases while inducing the production of an inflammatory polysaccharide, capable of enabling dendritic cells to generate pro-inflammatory cytokines such as tumor necrosis factor-alpha (TNF-α) (35), with a few studies confirming elevated levels of intestinal inflammatory cytokines, including TNF-α, in individuals with insomnia (4, 34). In patients with chronic insomnia, these pro-inflammatory bacteria are also responsible for disrupting brain activity. Further research is essential to understand other mechanisms of family Ruminococcaceae’s effect on insomnia.

Present studies indicate that the genus Lachnospiraceae UCG004 demonstrates a negative correlation with sleep efficiency and total sleep time (36). The abundance of the genus Lachnospiraceae is significantly increased in the gut of mice deprived of brief insomnia (37). In rats treated with gardenia for insomnia, the GM showed changes, including a reduction in the genus Lachnospiraceae (38). In a study that evaluated the GM characteristics of early and late sleepers, the “owls” exhibited an increase in the genus Lachnospiraceae (39). Lachnospira and Bacteroides serve as indicator bacteria to distinguish acute insomnia patients from healthy controls (4). Furthermore, the genus Lachnospiraceae is a significant producer of butyrate (40), which belongs to SCFAs and has been shown to be a source of sleep-and wakefulness-related signals. Genus Lachnospiraceae can affect sleep by means of SCFA metabolism. By utilizing resting-state functional magnetic resonance imaging (rs-fMRI), altered functional connectivity strength was apparent in the left superior parietal gyrus of patients with chronic insomnia when associated with the genus Lachnospiraceae (41). The abundance of genus Lachnospiraceae even increased in the GM of mice exposed to sleep fragmentation (SF). Genus Lachnospiraceae exhibits pro-inflammatory effects (42), and it can induce macrophage recruitment and promote the transfer of lipopolysaccharide (LPS) from the digestive tract to the bloodstream (43). This suggests that the genus Lachnospiraceae may cause inflammatory processes associated with SF by allowing microbial products to escape into the circulation. Consequently, targeting the genus Lachnospiraceae may become vital for treating insomnia.

Flavonifractors might contribute to *in vivo* inflammatory homeostasis by suppressing TNF-α expression in the inflammatory milieu (44), as well as mitigating antigen-induced Th2 immune responses (45). Excessive secretion of pro-inflammatory cytokines is one of the pathogenic causes of insomnia (46). Studies have shown that this genus delays the progression of diabetes by producing SCFAs. Despite this evidence, further research is needed on the protective potential of Flavonifractors against insomnia. No research has been conducted on Olsenella and insomnia. Olsenella has demonstrated the capacity to utilize fructo-oligosaccharides (FOS) (47). FOS are known as prebiotics that deliver beneficial health effects through the restructuring of the GM. Additionally, Olsenella can produce metabolites such as butyric acid, acetic acid, lactic acid, and succinic acid, potentially elucidating the genus’ protective mechanisms.

Reverse MR analysis indicates that an elevation in the GM genus Eggerthella may be linked to insomnia, which is in agreement with prior research. Additionally, insomnia may be connected to a decrease in the GM genus Holdemanella and order NB1n, and the exact mechanisms by which they influence sleep remain unknown.

This study boasts several noteworthy advantages over prior works. First, this study is the first MR study to focus on GM and insomnia. The recognition of specific gut bacteria as potential biomarkers can revolutionize insomnia diagnosis, moving away from subjective sleep logs to more objective, microbiota-based assessments. Insomnia exerts a considerable toll on patients’ quality of life and often necessitates treatment with hypnotics and sedatives. However, concerns surrounding drug side effects and addiction risk among certain patients persist. Thus, the GM pinpointed in this research might pave the way for innovative insomnia interventions. Adjusting the GM composition through dietary measures, probiotics, or alternative microbiome-focused therapies could provide new therapeutic avenues for insomnia. Such strategies might diminish the reliance on drug treatments, presenting a more integrative solution to insomnia management. Second, in contrast to previous observational studies, our genetic variant data was derived from the most extensive available GWAS meta-analysis, thus securing the effectiveness of instruments in the MR analysis. Such an approach aids in mitigating potential confounding influences.

Notwithstanding, a few constraints in our study merit discussion. First, our selection of GM instrumental variables (IVs) involved MibioGen study SNPs that were smaller than a threshold of 5 × 10^−8^, which proved inadequate for MR analysis. Consequently, the significance threshold for GM IVs was set at 1 × 10^−5^. While an F-statistic exceeding 10 was employed to counteract weak instrument bias, this threshold modification may engender potential biases in the MR estimates, potentially yielding erroneous outcomes. Subsequent research should consider validating these findings with alternative or more stringent instrumental variables to ascertain result reliability. Second, although the present analysis of the results demonstrated the effect of exposure on outcomes, the subgroup analyses were incomplete due to the lack of GWAS data, for example, separate subgroup analyses were not carried out for gender, geography, and age. To achieve a more rigorous level of evidence, comprehensive data collection is essential. Third, the research community still awaits direct mechanistic examinations supporting our findings. To acquire more explicit proof of the GM-insomnia correlation, further inquiries pertaining to the effects of these bacteria on inflammation, immune reactions, blood–brain barrier permeability, and brain maturation must be undertaken. Fourth, these findings pertain primarily to European cohorts. Variations in GM and genetic predispositions among diverse ethnic groups might modulate the association with insomnia. Hence, extrapolation to non-European populations warrants prudence. Finally, the number of SNPs involved in some analyses was too small, which made it impossible to conduct separate subgroup analyses. Additionally, the limited number of SNPs precluded the use of a fully IVW-based MR method or comprehensive sensitivity analyses, possibly leading to potential biases that went undetected.

To conclude, our study thoroughly investigated the feasible causal association of GM with insomnia. Our two-way MR investigation uncovered that the family Ruminococcaceae and the genus Lachnospiraceae UCG004 are prominent risk factors for insomnia, whereas the genera Flavonifractor and Olsenella may act as efficacious protective factors against this disorder. These outcomes warrant further scrutiny as they may emerge as promising gut biomarkers and novel therapeutic targets for insomnia.

## Data availability statement

The original contributions presented in the study are included in the article/Supplementary material, further inquiries can be directed to the corresponding author.

## Ethics statement

Ethical review and approval was not required for the study on human participants in accordance with the local legislation and institutional requirements. Written informed consent from the patients/participants or patients/participants’ legal guardian/next of kin was not required to participate in this study in accordance with the national legislation and the institutional requirements.

## Author contributions

JY: Conceptualization, Data curation, Formal analysis, Funding acquisition, Writing – original draft, Writing – review & editing. TS: Conceptualization, Data curation, Formal analysis, Investigation, Writing – original draft. YZ: Conceptualization, Data curation, Formal analysis, Writing – original draft. MJ: Conceptualization, Data curation, Formal analysis, Project administration, Writing – original draft. XY: Methodology, Project administration, Writing – review & editing. YL: Funding acquisition, Writing – review & editing. LC: Supervision, Validation, Visualization, Writing – review & editing.

## References

[1] Morin CM Drake CL Harvey AG Krystal AD Manber R Riemann D. Insomnia Disorder. Nat Rev Dis Primers. (2015) 1:15026. doi: 10.1038/nrdp.2015.26 27189779

[2] Amir I Bouvet P Legeay C Gophna U Weinberger A. Eisenbergiella tayi gen. nov., sp nov., isolated from human blood. Int J Syst Evol Microbiol. (2014) 64:907–14. doi: 10.1099/ijs.0.057331-0, PMID: 24282142

[3] Wang Z Wang Z Lu TS Chen WH Yan W Yuan K. The microbiota-gut-brain Axis in sleep disorders. Sleep Med Rev. (2022) 65:101691. doi: 10.1016/j.smrv.2022.101691 36099873

[4] Li YY Zhang B Zhou Y Wang DM Liu XC Li L. Gut microbiota changes and their relationship with inflammation in patients with acute and chronic insomnia. Nat Sci Sleep. (2020) 12:895–905. doi: 10.2147/nss.S271927, PMID: 33177907 PMC7652227

[5] Jackson ML Butt H Ball M Lewis DP Bruck D. Sleep quality and the treatment of intestinal microbiota imbalance in chronic fatigue syndrome: a pilot study. Sleep Sci. (2015) 8:124–33. doi: 10.1016/j.slsci.2015.10.001, PMID: 26779319 PMC4688574

[6] Greenland S. An introduction to instrumental variables for epidemiologists. Int J Epidemiol. (2000) 29:722:1102. doi: 10.1093/oxfordjournals.ije.a019909 10922351

[7] Smith GD Ebrahim S. 'Mendelian Randomization': can genetic epidemiology contribute to understanding environmental determinants of disease? Int J Epidemiol. (2003) 32:1–22. doi: 10.1093/ije/dyg070, PMID: 12689998

[8] Skrivankova VW Richmond RC Woolf BAR Davies NM Swanson SA VanderWeele TJ. Strengthening the reporting of observational studies in epidemiology using Mendelian randomisation (Strobe-Mr): explanation and elaboration. BMJ. (2021) 375:n2233. doi: 10.1136/bmj.n2233, PMID: 34702754 PMC8546498

[9] Skrivankova VW Richmond RC Woolf BAR Yarmolinsky J Davies NM Swanson SA. Strengthening the reporting of observational studies in epidemiology using Mendelian randomization: the Strobe-Mr statement. JAMA. (2021) 326:1614–21. doi: 10.1001/jama.2021.18236, PMID: 34698778

[10] Kurilshikov A Medina-Gomez C Bacigalupe R Radjabzadeh D Wang J Demirkan A. Large-scale association analyses identify host factors influencing human gut microbiome composition. Nat Genet. (2021) 53:156–65. doi: 10.1038/s41588-020-00763-1, PMID: 33462485 PMC8515199

[11] Kurki MI Karjalainen J Palta P Sipila TP Kristiansson K Donner KM. Finngen provides genetic insights from a well-Phenotyped isolated population. Nature. (2023) 613:508–18. doi: 10.1038/s41586-023-05837-8, PMID: 36653562 PMC9849126

[12] Burgess S Thompson SG. Bias in causal estimates from Mendelian randomization studies with weak instruments. Stat Med. (2011) 30:1312–23. doi: 10.1002/sim.4197 21432888

[13] Bowden J Del Greco MF Minelli C Smith GD Sheehan NA Thompson JR. Assessing the suitability of summary data for two-sample Mendelian randomization analyses using Mr-Egger regression: the role of the I-2 statistic. Int J Epidemiol. (2016) 45:1961–74. doi: 10.1093/ije/dyw220, PMID: 27616674 PMC5446088

[14] Burgess S Dudbridge F Thompson SG. Combining information on multiple instrumental variables in Mendelian randomization: comparison of allele score and summarized data methods. Stat Med. (2016) 35:1880–906. doi: 10.1002/sim.6835, PMID: 26661904 PMC4832315

[15] Burgess S Thompson SG. Interpreting findings from Mendelian randomization using the Mr-Egger method (Vol 32, Pg 377, 2017). Eur J Epidemiol. (2017) 32:391–2. doi: 10.1007/s10654-017-0276-5, PMID: 28527048 PMC5506233

[16] Tan JS Hua L. Genetic predisposition between Covid-19 and three cardio-cerebrovascular diseases: a bidirectional, two-sample Mendelian randomization study. Chest. (2022) 161:A55. doi: 10.1016/j.chest.2021.12.086

[17] Verbanck M Chen C Neale B Ron D. Detection of widespread horizontal pleiotropy in causal relationships inferred from Mendelian randomization between complex traits and diseases. Eur J Hum Genet. (2019) 27:854–5. doi: 10.1038/s41588-018-0099-7 PMC6083837 29686387

[18] Hemani G Zhengn J Elsworth B Wade KH Haberland V Baird D. The Mr-Base platform supports systematic causal inference across the human phenome. elife. (2018) 7:34408. doi: 10.7554/eLife.34408, PMID: 29846171 PMC5976434

[19] Gao T Wang ZX Dong YL Cao J Lin RT Wang XT. Role of melatonin in sleep deprivation-induced intestinal barrier dysfunction in mice. J Pineal Res. (2019) 67:e12574. doi: 10.1111/jpi.12574, PMID: 30929267

[20] Dauvilliers Y Zammit G Fietze I Mayleben D Kinter DS Pain S. Daridorexant, a new dual orexin receptor antagonist to treat insomnia disorder. Ann Neurol. (2020) 88:647–51. doi: 10.1002/ana.25801 31953863

[21] Cummings JH Pomare EW Branch WJ Naylor CP Macfarlane GT. Short chain fatty acids in human large intestine, portal. Hepat Venous Blood Gut. (1987) 28:1221–7. doi: 10.1136/gut.28.10.1221, PMID: 3678950 PMC1433442

[22] Heath ALM Haszard JJ Galland BC Lawley B Rehrer NJ Drummond LN. Association between the Faecal short-chain fatty acid propionate and infant sleep. Eur J Clin Nutr. (2020) 74:1362–5. doi: 10.1038/s41430-019-0556-0, PMID: 31969698

[23] Magzal F Even C Haimov I Agmon M Asraf K Shochat T. Associations between fecal short-chain fatty acids and sleep continuity in older adults with insomnia symptoms. Sci Rep. (2021) 11:5. doi: 10.1038/s41598-021-83389-5 33603001 PMC7893161

[24] Szentirmai E Millican NS Massie AR Kapas L. Butyrate, a metabolite of intestinal Bacteria, enhances sleep. Sci Rep. (2019) 9:7035. doi: 10.1038/s41598-019-43502-1, PMID: 31065013 PMC6504874

[25] Bonaz B Bazin T Pellissier S. The Vagus nerve at the Interface of the microbiota-gut-brain Axis. Front Neurosci. (2018) 12:49. doi: 10.3389/fnins.2018.00049 29467611 PMC5808284

[26] Neufeld KAM Bienenstock J Bharwani A Champagne-Jorgensen K Mao YK West C. Oral selective serotonin reuptake inhibitors activate Vagus nerve dependent gut-brain Signalling. Sci Rep. (2019) 9:8. doi: 10.1038/s41598-019-50807-8, PMID: 31582799 PMC6776512

[27] Wang Z Chen WH Li SX He ZM Zhu WL Ji YB. Gut microbiota modulates the inflammatory response and cognitive impairment induced by sleep deprivation. Mol Psychiatry. (2021) 26:6277–92. doi: 10.1038/s41380-021-01113-1, PMID: 33963281

[28] Burgos I Richter L Klein T Fiebich B Feige B Lieb K. Increased nocturnal Interleukin-6 excretion in patients with primary insomnia: a pilot study. Brain Behav Immun. (2006) 20:246–53. doi: 10.1016/j.bbi.2005.06.007, PMID: 16084689

[29] Liu BD Lin WF Chen SJ Xiang T Yang YF Yin YL. Gut microbiota as an objective measurement for auxiliary diagnosis of insomnia disorder (Vol 10, 1770, 2019). Front Microbiol. (2020) 11:510. doi: 10.3389/fmicb.2020.00510, PMID: 31456757 PMC6701205

[30] Feng Y Fu SS Li C Ma XF Wu YF Chen F. Interaction of gut microbiota and brain function in patients with chronic insomnia: a regional homogeneity study. Front Neurosci. (2022) 15:843. doi: 10.3389/fnins.2021.804843, PMID: 35069107 PMC8766814

[31] Leone V Gibbons SM Martinez K Hutchison AL Huang EY Cham CM. Effects of diurnal variation of gut microbes and high-fat feeding on host circadian clock function and metabolism. Cell Host Microbe. (2015) 17:681–9. doi: 10.1016/j.chom.2015.03.006, PMID: 25891358 PMC4433408

[32] Poroyko VA Carreras A Khalyfa A Khalyfa AA Leone V Peris E. Chronic sleep disruption alters gut microbiota, induces systemic and adipose tissue inflammation and insulin resistance in mice. Sci Rep. (2016) 6:405. doi: 10.1038/srep35405, PMID: 27739530 PMC5064361

[33] Zhou J Wu XL Li ZL Zou Z Dou SW Li G. Alterations in gut microbiota are correlated with serum metabolites in patients with insomnia disorder. Front Cell Infect Microbiol. (2022) 12:662. doi: 10.3389/fcimb.2022.722662, PMID: 35252021 PMC8892143

[34] Biddle A Stewart L Blanchard J Leschine S. Untangling the genetic basis of Fibrolytic specialization by Lachnospiraceae and Ruminococcaceae in diverse gut communities. Diversity. (2013) 5:627–40. doi: 10.3390/d5030627

[35] Henke MT Kenny DJ Cassilly CD Vlamakis H Xavier RJ Clardy J. *Ruminococcus Gnavus*, a member of the human gut microbiome associated with Crohn's disease, produces an inflammatory polysaccharide. Proc Natl Acad Sci U S A. (2019) 116:12672–7. doi: 10.1073/pnas.1904099116, PMID: 31182571 PMC6601261

[36] Smith RP Easson C Lyle SM Kapoor R Donnelly CP Davidson EJ. Gut microbiome diversity is associated with sleep physiology in humans. PLoS One. (2019) 14:e0222394. doi: 10.1371/journal.pone.0222394, PMID: 31589627 PMC6779243

[37] El Aidy S Bolsius YG Raven F Havekes R. A brief period of sleep deprivation leads to subtle changes in mouse gut microbiota. J Sleep Res. (2020) 29:e12920. doi: 10.1111/jsr.12920, PMID: 31515894 PMC7757181

[38] Liu D Wang Q Li Y Yuan Z Liu Z Guo J. Fructus Gardeniae ameliorates anxiety-like behaviors induced by sleep deprivation via regulating hippocampal metabolomics and gut microbiota. Front Cell Infect Microbiol. (2023) 13:1167312. doi: 10.3389/fcimb.2023.1167312, PMID: 37377643 PMC10291143

[39] Carasso S Fishman B Lask LS Shochat T Geva-Zatorsky N Tauber E. Metagenomic analysis reveals the signature of gut microbiota associated with human Chronotypes. FASEB J. (2021) 35:e22011. doi: 10.1096/fj.202100857RR, PMID: 34695305

[40] Leth ML Pichler MJ Abou HM. Butyrate-producing colonic Clostridia: picky glycan utilization specialists. Essays Biochem. (2023) 67:415–28. doi: 10.1042/ebc20220125, PMID: 36350044

[41] Chen ZW Feng Y Li SM Hua KL Fu SS Chen F. Altered functional connectivity strength in chronic insomnia associated with gut microbiota composition and sleep efficiency. Front Psych. (2022) 13:13. doi: 10.3389/fpsyt.2022.1050403, PMID: 36483137 PMC9722753

[42] Nakanishi Y Sato T Ohteki T. Commensal gram-positive Bacteria initiates colitis by inducing monocyte/macrophage mobilization. Mucosal Immunol. (2015) 8:152–60. doi: 10.1038/mi.2014.53, PMID: 24938744

[43] Kameyama K Itoii K. Intestinal colonization by a Lachnospiraceae bacterium contributes to the development of diabetes in obese mice. Microbes Environ. (2014) 29:427–30. doi: 10.1264/jsme2.ME14054, PMID: 25283478 PMC4262368

[44] Mikami A Ogita T Namai F Shigemori S Sato T Shimosato T. Oral Administration of *Flavonifractor Plautii* Attenuates Inflammatory Responses in obese adipose tissue. Mol Biol Rep. (2020) 47:6717–25. doi: 10.1007/s11033-020-05727-6, PMID: 32808115

[45] Ogita T Yamamoto Y Mikami A Shigemori S Sato T Shimosato T. Oral administration of *Flavonifractor Plautii* strongly suppresses Th2 immune responses in mice. Front Immunol. (2020) 11:11. doi: 10.3389/fimmu.2020.00379, PMID: 32184789 PMC7058663

[46] Zielinski MR Gibbons AJ. Neuroinflammation, sleep, and circadian rhythms. Front Cell Infect Microbiol. (2022) 12:3096. doi: 10.3389/fcimb.2022.853096, PMID: 35392608 PMC8981587

[47] Mao BY Li DY Zhao JX Liu XM Gu ZN Chen YQ. Metagenomic insights into the effects of Fructo-oligosaccharides (Fos) on the composition of fecal microbiota in mice. J Agric Food Chem. (2015) 63:856–63. doi: 10.1021/jf505156h, PMID: 25598242

