# Supplementary material for: A bidirectional Mendelian randomization study investigating the causal role between gut microbiota and insomnia
Source: Front Neurol. 2023 Dec 7;14:1277996. doi: 10.3389/fneur.2023.1277996 (PMC10740168; doi:10.3389/fneur.2023.1277996)
Supplement: Supplementary file 3 [file Data_Sheet_1.docx]

Supplementary Material

**A Bidirectional Mendelian Randomization Study Investigating the Causal Role Between Gut Microbiota and Insomnia**

**Jie. Yang^1^, Tengfei. Su^1^, Yating. Zhang^2，^Menghan. Jia^1^, Xiang. Yin^1^, Yue. Lang^1^, Li. Cui***

*** Correspondence:** Li. Cui: email: [lcui@jlu.edu.cn](mailto:lcui@jlu.edu.cn)

# Supplementary Data

**Supplementary Table S1.** **The Association Between GM and insomnia. Sheet1:** Characteristics of the genetic instrument variables for GM in the Mendelian randomization study at level P < 1 × 10^–5^. **Sheet 2:** MR analysis of GM and insomnia risk. **Sheet 3:** Heterogeneity analysis of GM and insomnia risk. **Sheet 4:** MR Egger intercept analysis of the association between GM and insomnia risk.

**Supplementary Table S2.** **The Association Between insomnia and GM. Sheet1:** Characteristics of the genetic instrument variables for insomnia in the Mendelian randomization study at level P < 5 × 10^–6^. **Sheet 2:** MR analysis of genetically predicted insomnia and GM. **Sheet 3:** Heterogeneity analysis of genetically predicted insomnia and GM. **Sheet 4:** MR Egger intercept analysis of the genetically predicted insomnia and GM.

# Supplementary Figures and Tables

## Supplementary Figures

**Supplementary Figure 1: The result of reverse MR analysis.** (A: Forest plot of MR analysis of the causal relationship between insomnia and order NB1n, B: MR leave-one-out sensitivity analysis to assess the robustness of the causal relationship between insomnia and order NB1n, C: Scatter plot to visualize causal effect of insomnia on order NB1n, D: Funnel plot of the causal relationship between insomnia and order NB1n as assessed by MR analysis)

**Supplementary Figure 2: The result of reverse MR analysis.** (A: Forest plot of MR analysis of the causal relationship between insomnia and genus Holdemanella, B: MR leave-one-out sensitivity analysis to assess the robustness of the causal relationship between insomnia and genus Holdemanella, C: Scatter plot to visualize causal effect of insomnia on genus Holdemanella, D: Funnel plot of the causal relationship between insomnia and genus Holdemanella as assessed by MR analysis)

**Supplementary Figure 3: The result of reverse MR analysis.** (A: Forest plot of MR analysis of the causal relationship between insomnia and genus Eggerthella, B: MR leave-one-out sensitivity analysis to assess the robustness of the causal relationship between insomnia and genus Eggerthella, C: Scatter plot to visualize causal effect of insomnia on genus Eggerthella, D: Funnel plot of the causal relationship between insomnia and genus Eggerthella as assessed by MR analysis)
