# Supplementary figures and images for: A bidirectional Mendelian randomization study investigating the causal role between gut microbiota and insomnia
Source: Front Neurol. 2023 Dec 7;14:1277996. doi: 10.3389/fneur.2023.1277996 (PMC10740168; doi:10.3389/fneur.2023.1277996)

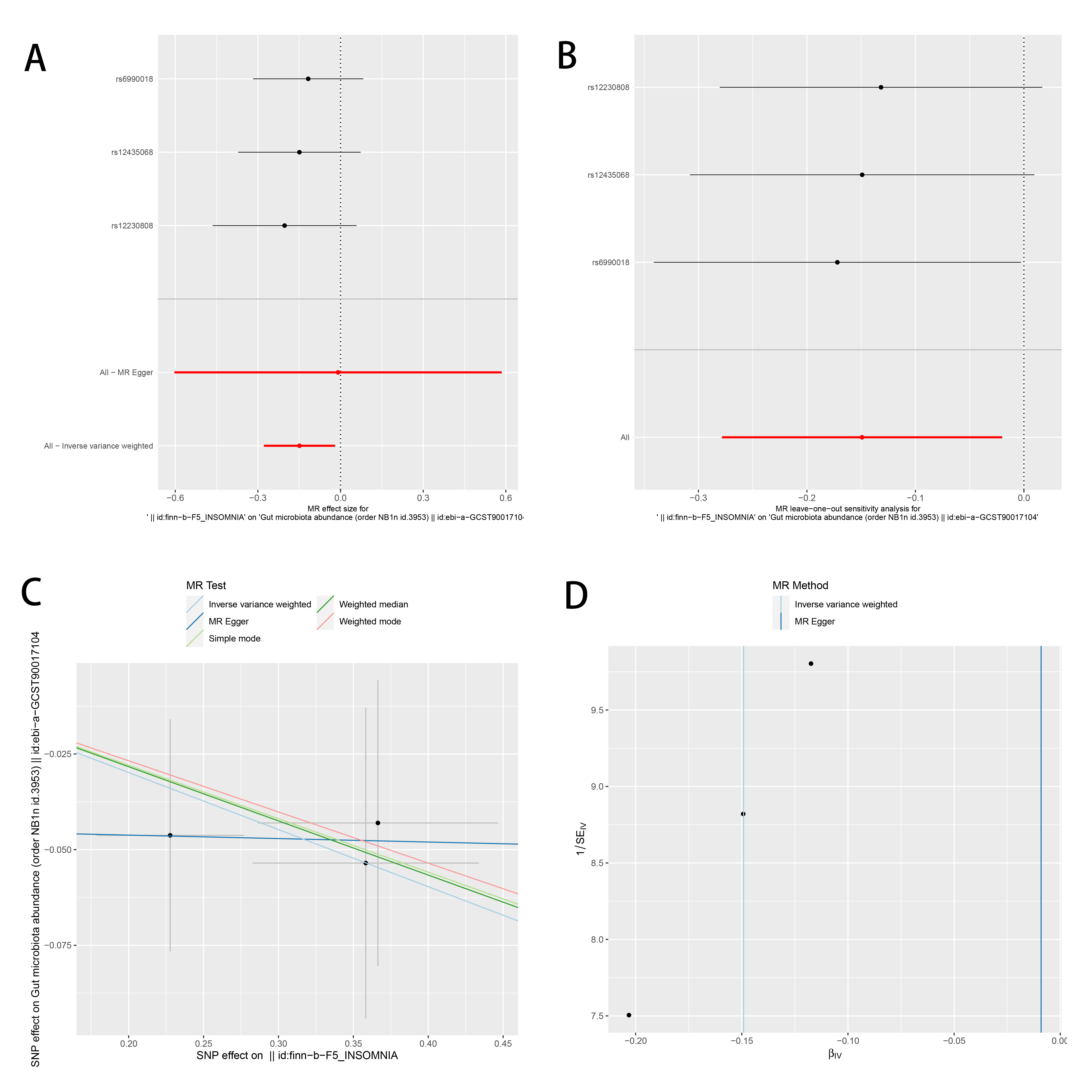

Supplement: Supplementary file 4 [file Image_1.jpeg]

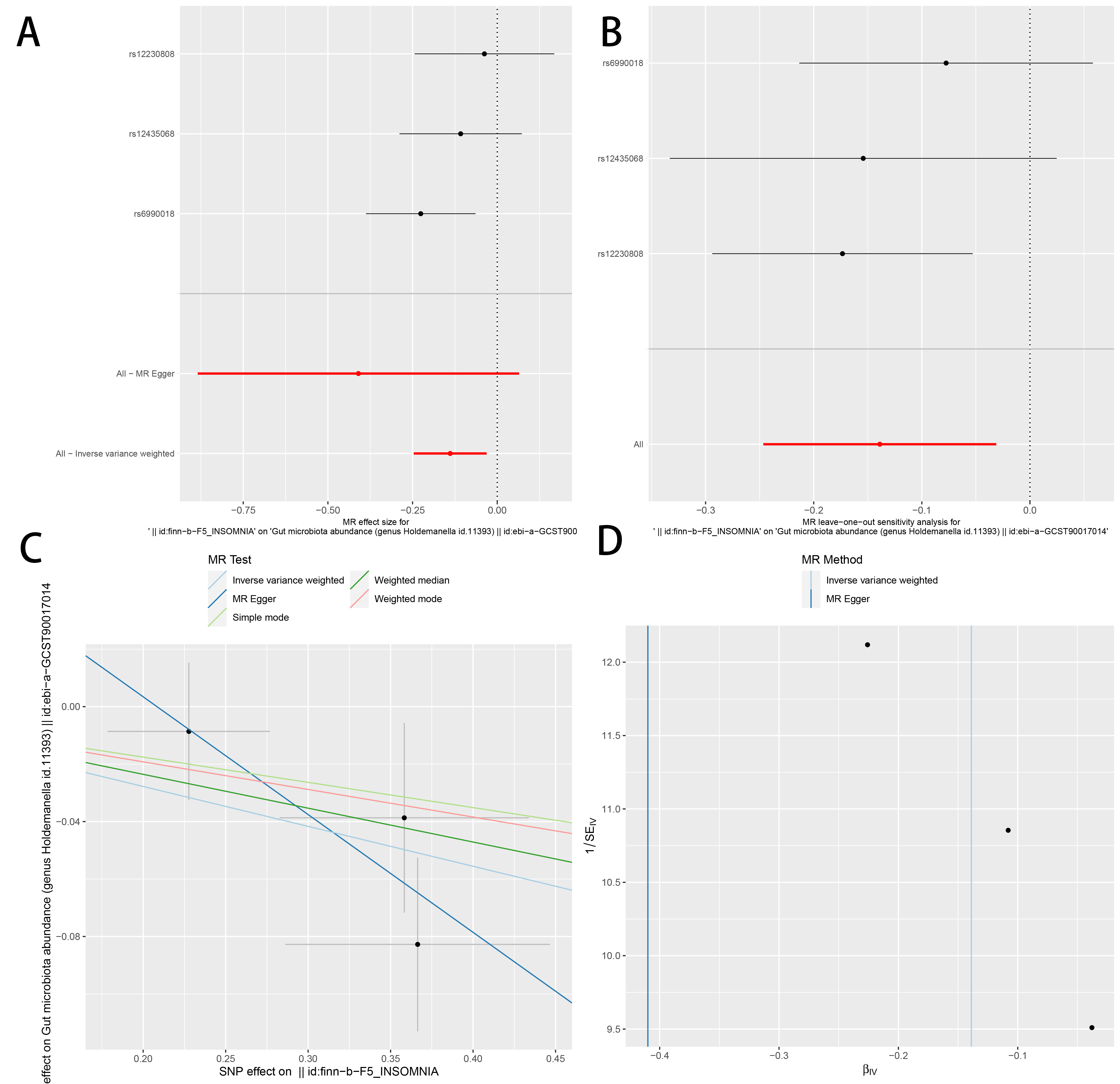

Supplement: Supplementary file 5 [file Image_2.jpeg]

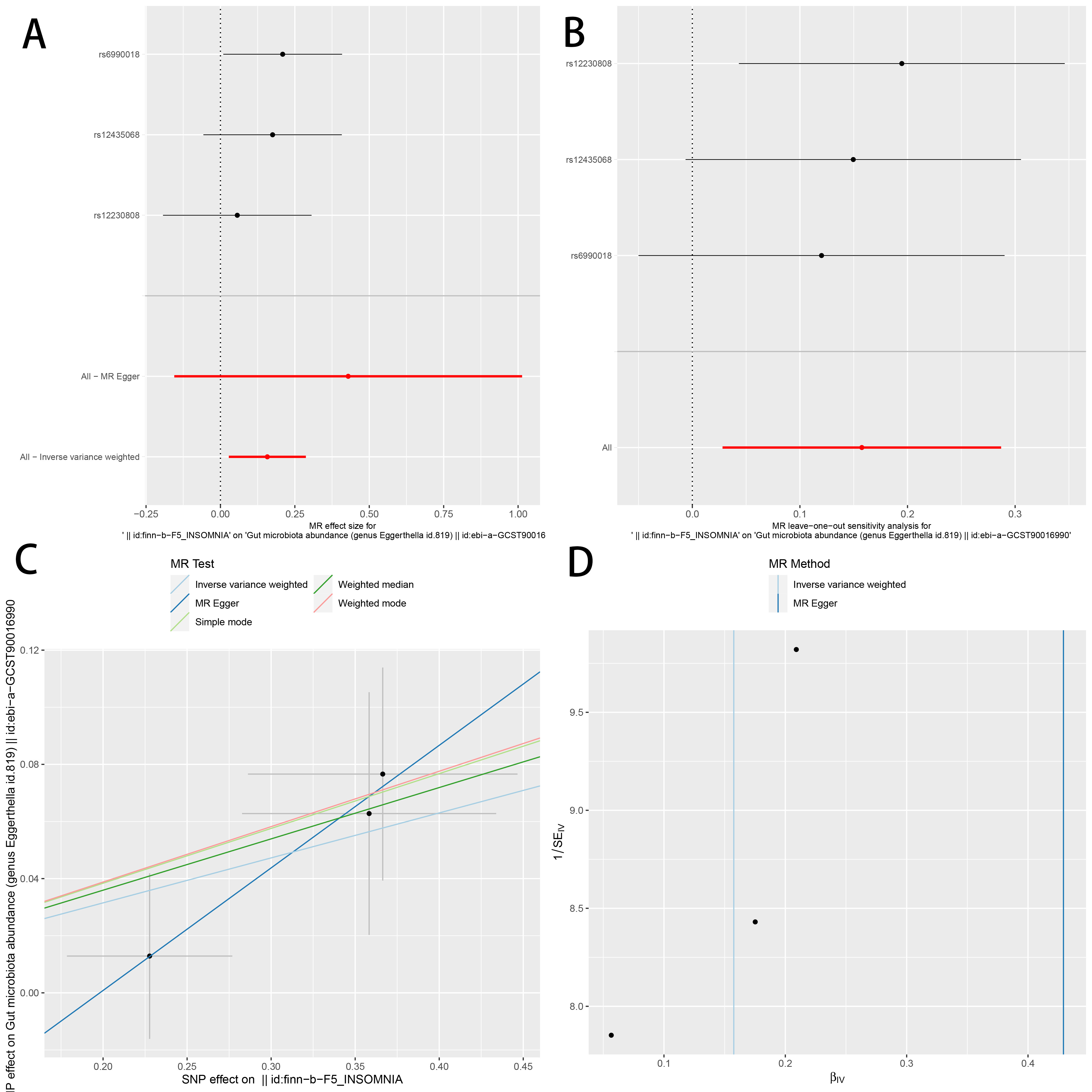

Supplement: Supplementary file 6 [file Image_3.jpeg]
